# *Shewanella oneidensis* Hfq promotes exponential phase growth, stationary phase culture density, and cell survival

**DOI:** 10.1186/1471-2180-13-33

**Published:** 2013-02-08

**Authors:** Christopher M Brennan, Meghan L Keane, Taylor M Hunt, Matthew T Goulet, Nicholas Q Mazzucca, Zachary Sexton, Taylor Mezoian, Katherine E Douglas, Jessica M Osborn, Brett J Pellock

**Affiliations:** 1 Department of Biology, Providence College, Providence, RI, USA

**Keywords:** *Shewanella oneidensis*, Hfq, Metal reduction, Oxidative stress, Stationary phase survival

## Abstract

**Background:**

Hfq is an RNA chaperone protein that has been broadly implicated in sRNA function in bacteria. Here we describe the construction and characterization of a null allele of the gene that encodes the RNA chaperone Hfq in *Shewanella oneidensis* strain MR-1, a dissimilatory metal reducing bacterium.

**Results:**

Loss of *hfq* in *S. oneidensis* results in a variety of mutant phenotypes, all of which are fully complemented by addition of a plasmid-borne copy of the wild type *hfq* gene. Aerobic cultures of the *hfq∆ * mutant grow more slowly through exponential phase than wild type cultures, and *hfq∆ * cultures reach a terminal cell density in stationary phase that is ~2/3 of that observed in wild type cultures. We have observed a similar growth phenotype when the *hfq∆ * mutant is cultured under anaerobic conditions with fumarate as the terminal electron acceptor, and we have found that the *hfq∆ * mutant is defective in Cr(VI) reduction. Finally, the *hfq∆ * mutant exhibits a striking loss of colony forming units in extended stationary phase and is highly sensitive to oxidative stress induced by H_2_O_2_ or methyl viologen (paraquat).

**Conclusions:**

The *hfq* mutant in *S. oneidensis* exhibits pleiotropic phenotypes, including a defect in metal reduction. Our results also suggest that *hfq* mutant phenotypes in *S. oneidensis* may be at least partially due to increased sensitivity to oxidative stress.

## Background

Hfq is an RNA chaperone broadly implicated in sRNA function in many bacteria. Hfq interacts with and stabilizes many sRNAs, and it is thought to help promote sRNA-mRNA target interactions [1,2]. Hfq protein monomers form a homohexameric ring that is thought to be the most active form of the protein [3,4]. Much of what is known about Hfq function is drawn from studies of loss of function alleles of *hfq* in bacteria including *Escherichia coli*[5], *Salmonella typhimurium*[6], and *Vibrio cholerae*[7]. A common *hfq* mutant phenotype is slow growth through exponential phase. However, loss of *hfq* function usually results in an array of mutant phenotypes, many of which are bacterium-specific. For example, *E. coli hfq* mutants exhibit slow growth *in vitro*[5], survive poorly in stationary phase, and are sensitive to both H_2_O_2_ and hyperosmotic conditions [8]. In contrast, *hfq* mutants in *Vibrio cholerae* grow reasonably well *in vitro* (though they exhibit impaired growth in a mouse infection model), survive normally in stationary phase, and are fully resistant to both H_2_O_2_ and hyperosmotic conditions [7]. Since many of the sRNAs that have been characterized require Hfq for their function, perhaps it is not surprising that loss of Hfq compromises a wide array of cellular processes. However, the fact that *hfq* mutations in different bacteria produce distinct phenotypes suggests distinct evolutionary roles for both Hfq and sRNAs in these divergent bacteria.

*Shewanella oneidensis* is a Gram-negative γ-Proteobacterium that is a facultative anaerobe found in a wide range of environments. *S. oneidensis* is a member of a class of bacteria known as the dissimilatory metal-reducing bacteria (DMRB). Under anaerobic conditions, *S. oneidensis* has the ability to utilize an impressively wide range of both organic and metallic terminal electron acceptors. These metallic terminal electron acceptors include Cr(VI), Fe(III), Mn(III) and (IV), and U(VI) [9,10]. The ability to mitigate the toxicity of soluble Cr(VI) and U(VI) by reduction to insoluble oxides of Cr(III) and U(IV), respectively, makes *Shewanella* an attractive potential bioremediating organism. In addition, the ability to deliver electrons to the extracellular environment allows *Shewanella* to generate electrical current in microbial fuel cells [11]. Because the transition between aerobic and anaerobic metabolism is likely to occur frequently in nature, it is probable that sRNAs play a role in the transition between these metabolic states in *S. oneidensis*.

To gain insight into the functions of Hfq in *S. oneidensis*, we have constructed and characterized a null allele of the *hfq* gene. The *hfq∆ * mutation in *S. oneidensis* is pleiotropic, resulting in defects in aerobic growth and greatly reduced recovery of colony forming units (CFU) from stationary phase cultures. In addition, loss of *hfq* results in compromised anaerobic growth on fumarate and diminished capacity to reduce Cr(VI). Finally, we have found that the *S. oneidensis hfq∆ * mutant is highly sensitive to oxidative stress. Importantly, each of the *hfq* mutant phenotypes we have described is complemented by a plasmid-borne copy of the wild type *S. oneidensis hfq* gene, strongly suggesting that the mutant phenotypes we have observed are the result of the loss of *hfq* and not due to disruption of another gene. Our results suggest that Hfq in *S. oneidensis* is involved in both common and organism-specific regulatory processes. To our knowledge, this is the first characterization of an *hfq* mutant in a dissimilatory metal reducing bacterium.

## Methods

### Media and growth conditions

Aerobic cultures were grown in either LB (10g/L tryptone, 5g/L yeast extract, 10g/L NaCl) or a modified version of the original M1 medium [9] with 30mM lactate as the electron donor. The modified M1 medium used in this study contains buffer/salts (3mM PIPES buffer, pH 7.0, 28mM NH_4_Cl, 1.34mM KCl, 4.4mM NaH_2_PO_4_, 125mM NaCl), vitamins [81.8nM D-biotin (vitamin B_7_), 45.3nM folic acid (vitamin B_9_), 486.4nM pyridoxine HCl (vitamin B_6_), 132.8nM riboflavin (vitamin B_2_), 133.6nM thiamine HCl (vitamin B_1_), 406.2nM nicotinic acid (vitamin B_3_), 209.8nM D-pantothenic acid, 0.74nM vitamin B_12_, 364.6nM p-aminobenzoic acid, 242.4nM lipoic acid], minerals [78.5μM nitriloacetic acid (trisodium salt), 249.1μM MgSO_4_ · 7 H_2_O, 29.6μM MnSO_4_ · 1 H_2_O, 171.1μM NaCl, 3.6μM FeSO_4_ · 7 H_2_O, 6.8μM CaCl_2_ · 2 H_2_O, 4.2μM CoCl_2_ · 6 H_2_O, 9.54μM ZnCl_2_, 0.4μM CuSO_4_ · 5 H_2_O, 0.21μM AlK(SO_4_)_2_ · 12 H_2_O, 1.61μM H_3_BO_3_, 1.24μM Na_2_MoO_4_ · 2 H_2_O, 1.01μM NiCl_2_ · 6 H_2_O, 0.76μM Na_2_WO_4_ · 2 H_2_O], and amino acids (135.9μM L-glutamic acid, 114.8μM L-arginine, 190.3μM DL-serine). Anaerobic cultures were grown in modified M1 medium with 30mM lactate as the electron donor and 30mM sodium fumarate as the electron acceptor. Anaerobic conditions in broth cultures were achieved by treating cultures in sealed test tubes using Oxyrase for Broth (Oxyrase, Inc., Mansfield, Ohio) as per the manufacturer’s instructions.

All *S. oneidensis* cultures were grown at 30°C, while *E. coli* cultures were grown at 37°C. Cultures containing both *E. coli* and *S. oneidensis* were grown at 30°C. Antibiotics were used at the following concentrations: Gentamicin (Gm): 5 μg/ml; Tetracycline (Tc): 10 μg/ml for *E. coli*; 1 μg/ml for *S. oneidensis*, [we used a lower concentration of Tc for selection of *S. oneidensis* than for *E. coli* because we found that the minimum inhibitory concentration (MIC) of Tc for *S. oneidensis* MR-1 is <1 μg/ml (data not shown)]; Kanamycin (Km): 25 μg/ml; Ampicillin (Amp): 100 μg/ml.

For growth curves, 5ml LB Km cultures of *S. oneidensis* strains were inoculated from frozen permanent stocks and aerobically outgrown overnight (10–12 hours). The overnight cultures were diluted in LB Km to an ABS_600_ ≅ 0.1 or in modified M1 Km to an ABS_600_ ≅ 0.025 and aerobically outgrown to log phase (ABS_600_ ≅ 0.4-0.8). These exponentially growing cultures were then diluted to an ABS_600_ ≅ 0.1 (LB Km) or to an ABS_600_ ≅ 0.025 (modified M1 Km). Aerobic cultures (15-20ml) were grown in 125mL Erlenmeyer flasks shaken at 250RPM. Anaerobic cultures (15ml) were grown in sealed test tubes without shaking. Culture densities (ABS_600_) were monitored spectrophotometrically, and culture titers (CFU/ml) were determined by plating serial dilutions of cultures on LB Km plates.

### Construction of the *S. oneidensis hfq∆ * mutant and *hfq* rescue construct

**Figure 1:**
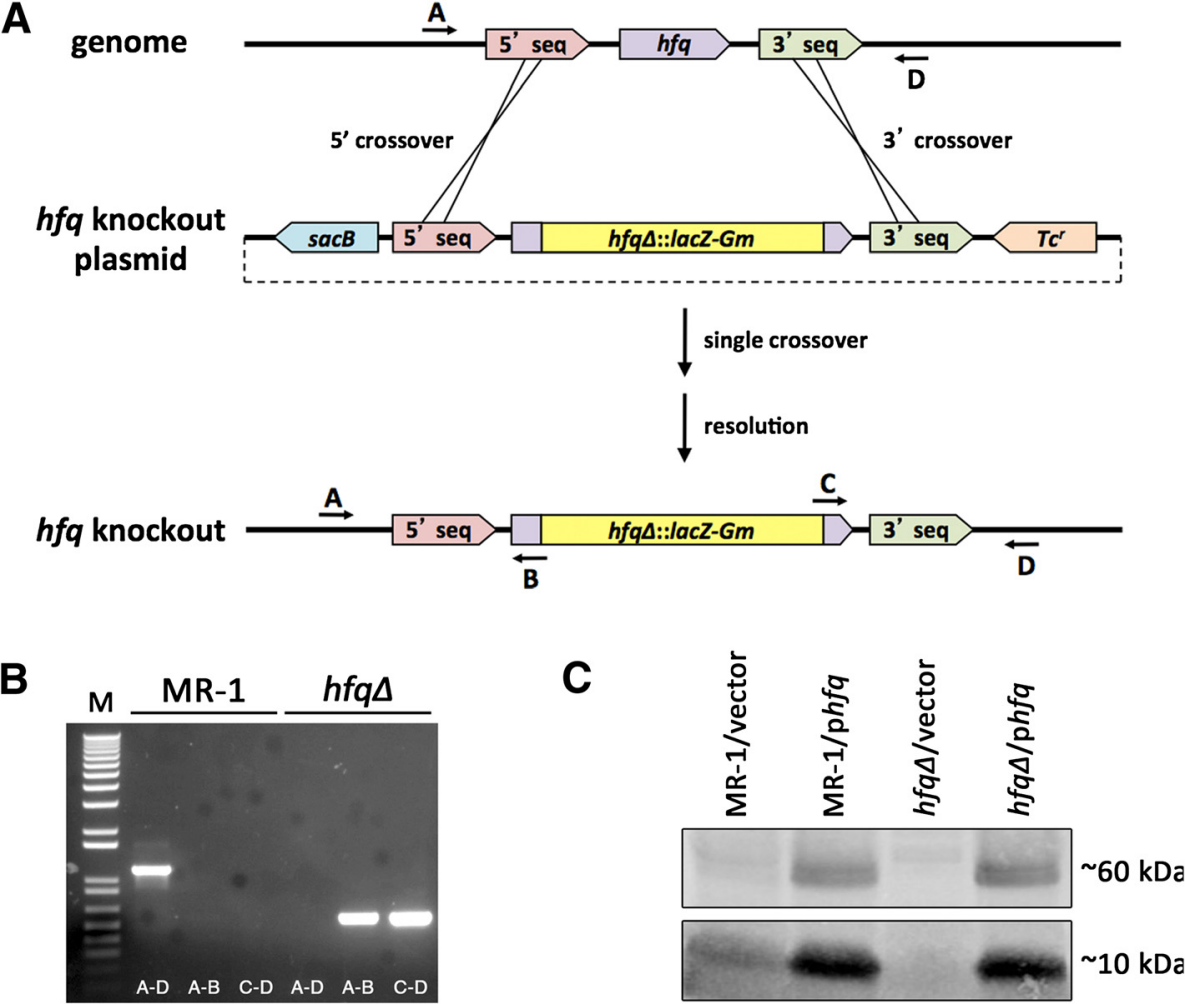
**Construction and verification of a null allele of the *Shewanella oneidensis *MR-1 *hfq *gene. **(**A**) Knockout strategy for the MR-1 *hfq* gene. Most of the *hfq* gene coding sequence (all but the first 9 codons and last 6 codons) was replaced with a cassette containing a promoterless *lacZ* gene and a gentamicin resistance marker. (**B**) Agarose gel of analytical PCR reactions using wild type MR-1 (lanes 2–4) or *hfq∆ * mutant (lanes 5–7) templates and primers A, B, C, and D (see Materials and Methods for primer sequences) indicated with arrows on the diagram in panel (A) The size standard (M) in lane 1 is 1kb plus DNA ladder (Invitrogen). (**C**) Western blot of SDS-PAGE-fractionated total protein from various *Shewanella* strains probed with a polyclonal antibody raised against *E. coli* Hfq protein. Lanes 1 and 2: MR-1 containing pBBR1-MCS-2 (vector) or *hfq* rescue construct (p*hfq*), respectively. Lanes 3 and 4: *hfq∆ * containing vector or p*hfq*, respectively. The antibody detects both putative Hfq monomers (~10kDa) as well as putative Hfq homohexamers (~60kDa).

To generate a null allele of *hfq* (So_0603 [12]) we deleted most of the *hfq* open reading frame and replaced it with a promoterless *lacZ*/gentamicin resistance gene cassette from pAB2001 [13]. We first PCR amplified a 5^′^ fragment using the primers GGCCCCGGGTAGAGCAAGGCTTTATTGATGAGGTAGC and GGCGCATGCGTCTTGTAAAGATTGCCCCTTAGCC and a 3’ fragment using the primers GGCGCATGCACGATATGCCAAGTGGCGAATAAGG and GGCGGTACCAGCTCGTTGGGCGAAAATATCCAAAATCAG. Following restriction (restriction endonucleases purchased from New England Biolabs, Ipswich, MA) of the 5^′^ PCR fragment with XmaI and SphI and restriction of the 3’ PCR fragment with SphI and KpnI, the two fragments were simultaneously ligated into pBSKS II + [14] that had been restricted with XmaI and KpnI. A 4.5kb SphI fragment from pAB2001 was then inserted into the SphI site of this plasmid to generate pBS-*hfq∆.* The XmaI-KpnI fragment from pBS-*hfq∆,* which contained the *lacZ*/gentamicin-disrupted *hfq* gene, was then cloned into XmaI/KpnI restricted pDMS197 [15], a R6K *ori* plasmid. The resulting plasmid, pDMS197-*hfq∆ * was transformed into *E. coli* SM10λ*pir*[16], mated into *S. oneidensis* MR-1 [9], and Gm^r^/Tc^r^ single crossover recombinants were isolated. Following growth in LB liquid without selection, cultures of these single crossovers were plated to LB plates containing Gm, 5% sucrose (w/v), and 0.1% NaCl (instead of omiting NaCl to increase the likelihood of isolating an *hfq* mutant in the event that loss of *hfq* resulted in cells sensitive to hypoosmotic conditions). Gm^r^ Suc^r^ Tc^s^*hfq∆ * mutant candidates were screened via PCR and DNA sequencing of the disrupted region. The sequence of the primers used for diagnostic PCR in Figure 1 are as follows: A (*hfq* 5’ diagnostic) - ATAATGTGGTGCAATTTGCC; B (*lacZ* 5’ out) - CGTTGTAAAACGACGGGATCG; C (*aacC1* 3’out) - GATGCACTTTGATATCGACCC; D (*hfq* 3’ diagnostic) - GAGTCCAACCACGCACTAGG. 

To generate an *hfq* rescue construct, we PCR amplified a 1.3kb genomic fragment containing the *S. oneidensis* MR-1 *hfq* coding sequence and ~1kb upstream of the *hfq* open reading frame. Based on *hfq* promoter analysis in *E. coli*, this fragment likely contains the native promoters for *S. oneidensis hfq*[17]. A PCR product was generated using the 5’ primer GGCAAGCTTCAGGAAAAACGGCTTTAGCTCTCG and the 3’ primer GGCGGTACCACTAAACCTTATTCGCCACTTGGC. Following restriction with HindIII and KpnI, this PCR product was ligated to HindIII/KpnI restricted pBBR-1MCS2 [18]. The resulting plasmid, pBBR1-*hfq*, was transformed into *E. coli* S17-1λ*pir*[19] and mated into *S. oneidensis* strains. In our hands, the pBBR1-MCS2 based vectors were stably maintained in *S. oneidensis* strains after 30 hours in LB Km cultures and after 120 hours in modified M1 Km cultures (data not shown).

### Western blot analyses

3ml aliquots of *S. oneidensis* cultures were pelleted in a microcentrifuge for 2’ at 20300 x *g*. Bacterial pellets were frozen at −80°C, thawed, and then treated with Bacterial Protein Extraction Reagent (B-PER) in the presence of 100μg/ml lysozyme, 5U/ml DNAse I, and 1X Halt Protease Inhibitor Cocktail. Protein was quantified using the Pierce BCA Protein Assay Kit as per manufacturers instructions (all reagents were obtained from Thermo Scientific, Rockford, IL).

For western blot analysis, 90μg of protein per lane was size fractionated at 4°C using Any kD Mini-PROTEAN TGX Precast Gels (Bio-Rad, Hercules, CA). Proteins were then transferred to an Immobilon-P^SQ^ PVDF membrane (EMD Millipore, Billerica, MA). Equivalent protein in different lanes was verified by Ponceau S staining of the membrane (data not shown). The membrane was blocked for 1 hour at room temperature using LI-COR Odyssey Blocking Buffer (LI-COR Biosciences, Lincoln, NE) and probed with a 1:5000 dilution of primary antibody, rabbit anti-*E. coli* Hfq [20] overnight at 4°C. The blot was washed 4 times for 5 minutes each with PBS-T and then probed with a 1:10000 dilution of goat anti-rabbit secondary antibody conjugated to IRDye 800CW Infrared Dye (LI-COR Biosciences, Lincoln, NE) for 45 minutes at room temperature (~22°C). The blot was washed with PBS-T 4 times for 5 minutes each and then rinsed with PBS to remove residual Tween 20. The blot was then imaged on a LI-COR Odyssey infrared scanner. Protein in Figure 1C was harvested from 24 hour old LB Km cultures. Older cultures consistently accumulated higher levels of Hfq protein, though our western blot results were consistent regardless of culture age at harvest; we never observed Hfq protein in the *hfq∆*/empty vector cultures (Figure 1C and data not shown).

### Chromium reduction assays

Chromium reduction assays were performed using a diphenylcarbazide-based quantitative, valence state specific, colorimetric assay for Cr(VI) [21]. Log phase cultures (ABS_600_ ≅ 0.5-0.8) grown in modified M1 medium were diluted to ABS_600_ ≅ 0.4 in modified M1 medium that had been prewarmed to 30°C. The cultures were transferred to sealed test tubes and treated for 30 minutes at 30°C with Oxyrase for Broth (Oxyrase, Inc., Mansfield, Ohio) to remove oxygen. Following addition of 100μM K_2_CrO_4_, cultures were incubated without shaking in a 30°C water bath in sealed test tubes. 1ml aliquots of cultures were periodically removed and added to 13mm borosilicate glass tubes containing 0.25ml of a 0.5% diphenylcarbazide solution in acetone and 2.5ml 0.28N HCl. Following vortexing, ABS_541_ values for individual samples were measured in a SPECTRONIC 20D+ spectrophotometer (Thermo Scientific, Rockford, IL).

### Oxidative stress assays

Overnight cultures grown in LB Km were diluted to an ABS_600_ ≅ 0.1. These cultures were outgrown for 2–3 hours to exponential phase (ABS_600_ ≅ 0.4-0.6) then diluted to an ABS_600_ ≅ 0.2. Following five minutes of aerobic growth, cultures were treated with H_2_O (mock), 0.4 mM H_2_O_2_ to induce peroxide stress, or 5 mM methyl-viologen (paraquat) to induce superoxide stress. Cultures were then grown aerobically for 15 minutes. Following treatment, each culture was serially diluted in triplicate in phosphate-buffered saline (PBS, pH 7.4). The dilutions were plated to LB Km plates within five minutes of harvest and grown overnight before scoring.

## Results

### Construction and verification of a null allele of *hfq* in *Shewanella oneidensis* MR-1

To study the roles played by the *hfq* gene in *Shewanella oneidensis*, we constructed a null allele of the putative *hfq* gene (So_0603) in *S. oneidensis* strain MR-1 [9,12]. To disrupt the *S. oneidensis hfq* gene, we generated a knockout construct in which we replaced most of the coding region of *hfq* with a cassette derived from pAB2001 [13] containing a promoterless *lacZ* gene and a gentamicin resistance marker (Figure 1A - see Materials and Methods for details). This knockout fragment was cloned into the Tc^r^*sacB*-counterselectable R6K *ori* suicide vector pDMS197 [15] and mobilized into *S. oneidensis* MR-1. Single crossovers of the *hfq* knockout plasmid into the MR-1 genome were isolated on the basis of both Gm resistance and ability to grow on modified M1 defined medium. Following PCR verification, LB cultures of Gm^r^ Tc^r^ single crossovers were outgrown in LB medium without antibiotic selection and then plated on LB agar containing Gm and 5% (w/v) sucrose. Elimination of the *hfq* gene in Suc^r^ Tc^s^ candidates was verified by PCR analyses (Figure 1B) and DNA sequencing analysis (data not shown). Western blotting demonstrated that the *hfq∆ * strain fails to produce Hfq protein (Figure 1C). Taken together, these data indicate that we have generated a null allele of *hfq* in *S. oneidensis*.

### The *Shewanella oneidensis hfq* mutant is defective in aerobic growth and exhibits reduced viable cell counts in stationary phase

**Figure 2:**
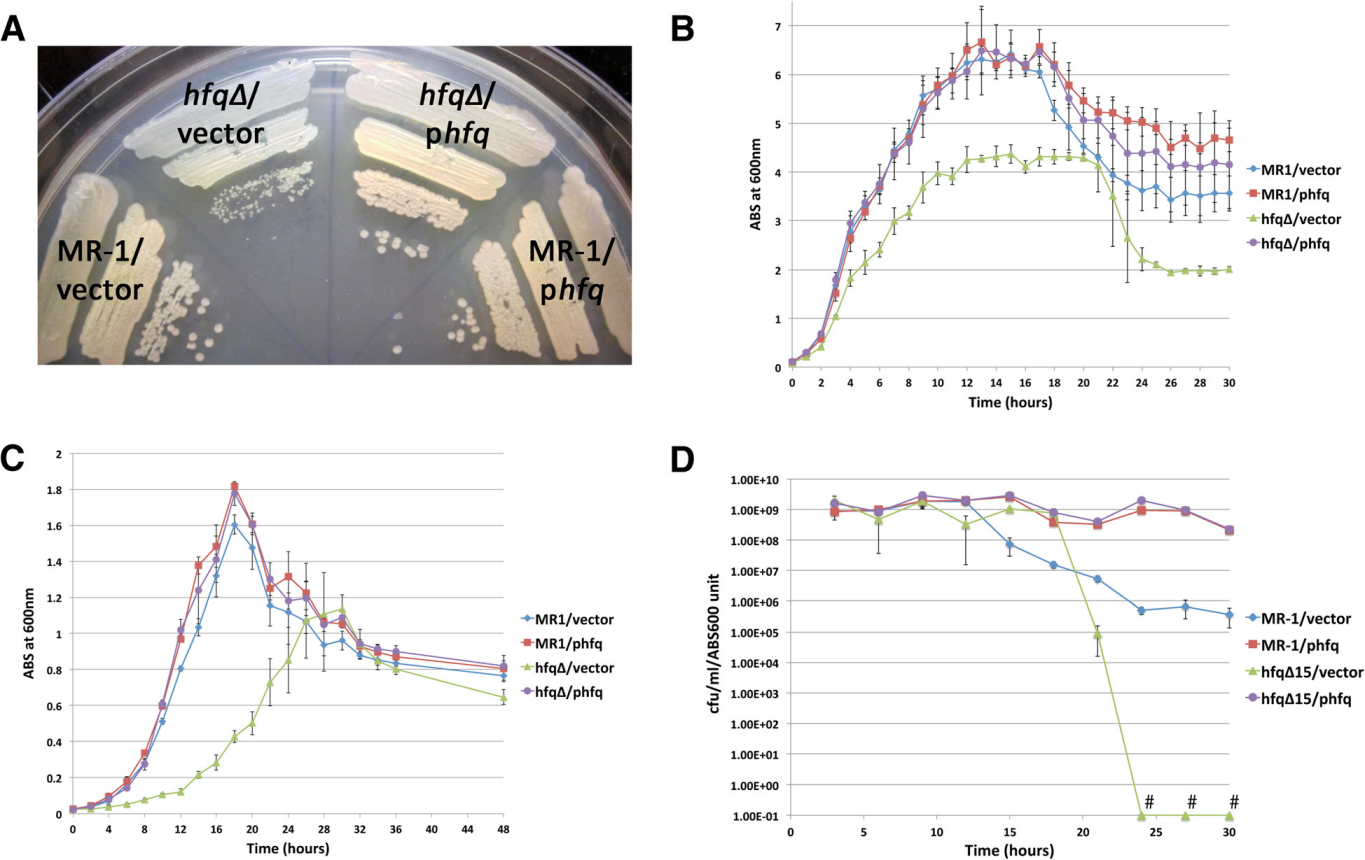
***Shewanella oneidensis *Hfq promotes exponential growth, terminal culture density, and viable cell counts in stationary phase. **Growth of MR-1/empty vector, MR-1/p*hfq*, *hfq∆ */empty vector, and *hfq∆ */p*hfq* on LB agar containing kanamycin (**A**), in LB liquid containing kanamycin (**B**), or in modified M1 defined medium containing kanamycin **(C)**. Plates in (**A**) were photographed after 24 hours of growth following inoculation from frozen permanent stocks. Three independent liquid cultures of each strain tracked in (**B-D**) were inoculated with log phase cultures grown in LB (**B** and **D**) or modified M1 medium (**C**). (**D**) Analysis of the relationship between viable cell counts (CFU/ml) and culture turbidity (ABS_600_) in LB cultures. Data points marked with “#” have CFU/ml/ABS_600_ values of zero. Error bars in panels (**B-D**) indicate a 99% confidence interval (P = 0.01).

Because mutations in the *hfq* gene compromise growth in many bacteria, we analyzed the growth properties of the *S. oneidensis hfq* null mutant. We characterized four strains: MR-1 containing pBBR1-MCS2 (hereafter referred to as empty vector), MR-1 containing pBBR1-*hfq* (pBBR1-MCS2 containing the wild type *hfq* gene under the control of its putative native promoter, hereafter referred to as p*hfq*), *hfq∆ * containing empty vector, and *hfq∆ * containing p*hfq*. Loss of the *hfq* gene resulted in a small colony phenotype on both LB agar plates (Figure 2A) and modified M1 defined medium plates (data not shown). The small colony phenotype of the *hfq* mutant was completely rescued by p*hfq*, but not by the empty vector alone (Figure 2A). The growth phenotype of wild type MR-1 cells containing the p*hfq* rescue plasmid was indistinguishable from MR-1 cells containing the empty vector (Figure 2A), suggesting that additional, plasmid-borne copies of *hfq* that result in higher Hfq protein levels than found in wild type cells (Figure 1C) do not significantly affect the growth of *S. oneidensis* on solid media. Of note is that the *hfq* mutant colonies with empty vector never attain the same colony size as strains harboring wild type *hfq*, even after extended incubation (data not shown). 

To further characterize the nature of the growth defect in the *hfq* mutant, we compared the growth of the *hfq* mutant in aerobic liquid cultures to strains containing one or more wild type copies of the *hfq* gene (Figure 2B). When exponentially-growing cultures were diluted to late lag phase and outgrown beyond stationary phase, we consistently observed that the *hfq∆*/empty vector culture densities were significantly lower than those of the MR-1/empty vector cultures through exponential phase. In addition, the terminal cell densities of stationary phase *hfq∆*/empty vector cultures were significantly lower than the terminal cell densities of MR-1/empty vector cultures (Figure 2B). We also observed delayed growth during exponential phase and lower terminal stationary phase densities in *hfq∆*/empty vector liquid cultures grown in modified M1, a defined medium (Figure 2C). The growth and terminal density defects of the *hfq* mutant in liquid cultures were completely rescued by p*hfq*, as the growth of the *hfq∆/*p*hfq* strain was indistinguishable from that of MR-1/empty vector in both LB (Figure 2B) and modified M1 (Figure 2C). Finally, extra copies of *hfq* that result in higher Hfq protein levels (Figure 1C) do not appear to alter the growth of *S. oneidensis* in liquid medium, as growth of MR-1/p*hfq* and *hfq∆/*p*hfq* cultures was indistinguishable from that of MR-1/empty vector cultures in LB and modified M1 media (Figures 2B and 2C).

To determine whether the relationships between spectrophotometric measurements of culture density and cell number were comparable between the strains used in our study, we determined the relationship between ABS_600_ values and viable cell counts for MR-1/empty vector, MR-1/p*hfq*, *hfq∆/*empty vector, and *hfq∆*/p*hfq* at various times during culture outgrowth. In both LB cultures (Figure 2D) and modified M1 cultures (data not shown), the relationship between ABS_600_ and colony forming units per ml (CFU/ml) was consistent for all four strains throughout exponential phase and until approximately mid-stationary phase. This indicates that the optical properties of the four strains characterized are highly similar at 600nm and that turbidity measurements are an accurate indicator of culture growth until mid stationary phase.

Intriguingly, we observed that the CFU/ml/ABS_600_ values for the four strains used in our studies diverged dramatically following mid-stationary phase (Figure 2D). We consistently found that *hfq∆/*empty vector cultures experienced a precipitous drop in CFU counts late in stationary phase. In most cases, culturable cell counts had dropped to zero CFU/ml by 30 hours. In contrast, MR-1/empty vector cultures were much more robust than *hfq∆ /*empty vector cultures, maintaining significant CFU counts, even after 30 hours of growth. The data presented in Figure 2D represents a typical result for an iteration of this experiment. It is worth noting, however, that the timing of the beginning of the reduction in CFU counts observed for the MR-1/empty vector strain and for the *hfq∆/*empty vector strain could vary by several hours between independent cultures, even parallel cultures simultaneously inoculated using the same preculture (data not shown). Furthermore, we also consistently observed that MR-1/p*hfq* and *hfq∆/*p*hfq* cultures, which contain more Hfq protein than wild type cultures at 24 hours (Figure 1C), retained significantly higher numbers of colony forming units compared to MR-1/empty vector cultures in extended stationary phase. Taken together, our loss-of-function and gain-of-function analyses demonstrate that Hfq promotes cell survival or culturability in extended stationary phase.

### The *hfq∆ * mutant is impaired in anaerobic growth and chromium reduction

**Figure 3:**
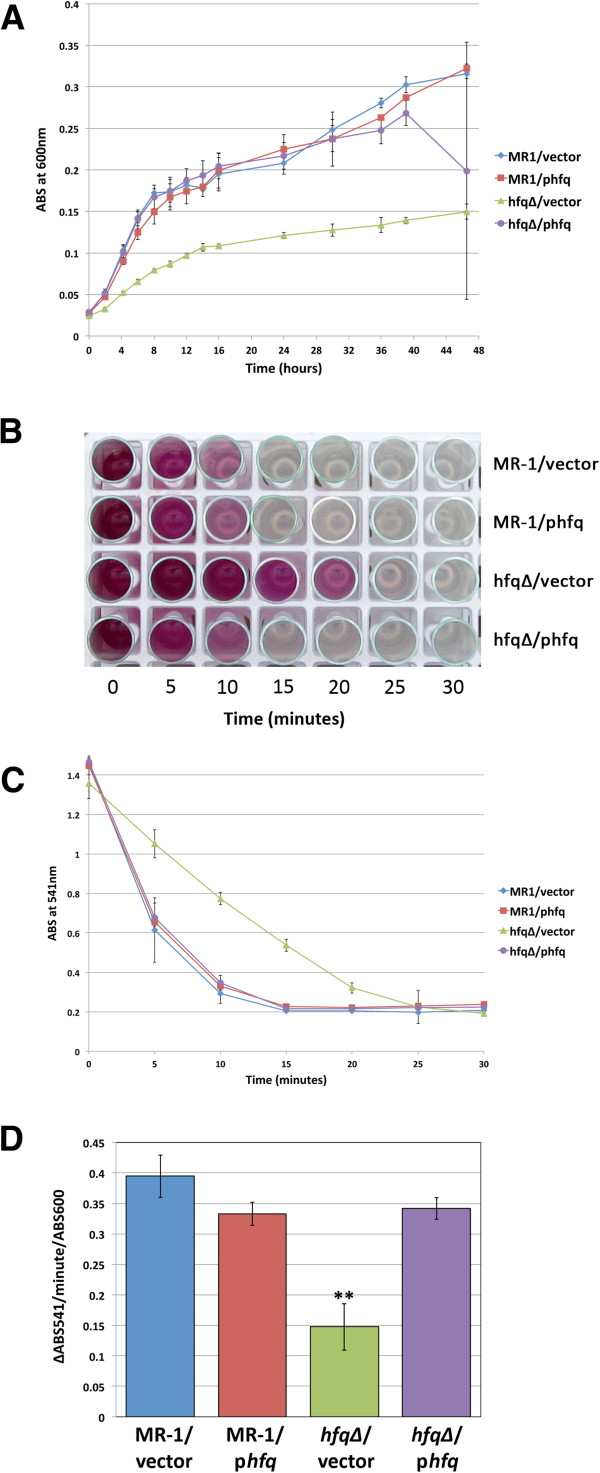
**The *hfq∆ *mutant is deficient in anaerobic respiration. **(**A**) Growth of MR-1/empty vector, MR-1/p*hfq*, *hfq∆ */empty vector, and *hfq∆ */p*hfq* under anaerobic conditions with fumarate as the terminal electron acceptor. Data presented is from three independent cultures. Error bars represent a 99% confidence interval (P = 0.01). (**B** and **C**) Results of chromium reduction assays. Chromium reduction/disappearance of Cr(VI) was assayed using the diphenylcarbazide method. Error bars represent a 99% confidence interval (P = 0.01). (**D**) Quantification of the rate of Cr(VI) reduction (expressed as the change in ABS_541_ per minute per ABS_600_) in the cultures tracked in (**C**) above during the first five minutes following the addition of chromium to anaerobic cultures. Error bars represent the standard deviation for triplicate cultures. ** indicates that the *hfq∆ */empty vector rate is statistically different from the other three strains (P < 0.002 for all three comparison in unpaired two-tailed Student’s T-tests).

To characterize the role of *S. oneidensis hfq* in anaerobic growth, we compared the growth kinetics of strains MR-1/empty vector, MR-1/p*hfq*, *hfq∆/*empty vector, and *hfq∆ */p*hfq* grown in modified M1 defined medium with fumarate as the terminal electron acceptor. Similar to the growth defects observed during aerobic growth, anaerobic *hfq∆ /*empty vector cultures grew more slowly during exponential phase and reached a lower terminal density than MR-1/empty vector cultures. (Figure 3A). The growth and terminal density defects of *hfq* mutant cultures in anaerobic modified M1 plus fumarate were completely rescued by p*hfq*, as the growth of the *hfq∆/*p*hfq* strain was indistinguishable from that of MR-1/empty vector (Figure 3A). Extra copies of *hfq* did not alter the ability of *S. oneidensis* to utilize fumarate as a terminal electron acceptor, as growth of MR-1/p*hfq* and *hfq∆/*p*hfq* cultures was very similar to that of MR-1/empty vector cultures (Figure 3A). 

To determine whether loss of *hfq* altered the ability of *S. oneidensis* to utilize chromium as a terminal electron acceptor, we measured the kinetics of Cr(VI) reduction by our four *hfq* strains using diphenylcarbazide, a reagent that binds to Cr(VI) and produces a purple color proportional to the amount of Cr(VI) in the sample [21]. In fully anaerobic cultures with no other electron acceptor present, metal reduction begins immediately upon addition of Cr(VI), and the rate of reduction is highest in the first five minutes following Cr(VI) addition. As the Cr(VI) is reduced, the assay results proceed from a deep purple color at early timepoints to a colorless solution at later timepoints, allowing quantification of the disappearance of Cr(VI) (Figure 3B). In our assays, the ABS_541_ values for the assay timepoints do not fall below ~0.2 because of the absorbance contribution of the cells at 541nm (data not shown). Though all strains tested eventually reduced all of the detectable Cr(VI), we found that the *hfq∆ * mutant is significantly slower in reducing Cr(VI) and takes nearly twice as long to utilize all available Cr(VI) as strains containing wild type *hfq* (Figures 3B and 3C). In addition, the rate of Cr(VI) reduction (∆ABS_541_) per minute per ABS_600_ unit during the first five minutes of metal reduction for the *hfq∆*/empty vector strain was less than half that of strains containing at least one copy of wild type *hfq* (Figure 3D).

To be certain that the Cr(VI) reduction defect observed in the *hfq∆*/empty vector strain was due to a defect in metal reduction and not death of cells due to an increased sensitivity to Cr, we measured the CFU/ml present in cultures of all four strains both before and after the 30 minute chromium reduction assay. We found no significant differences in the CFU/ml values measured before and after the assay for any of the four strains used in our experiments (data not shown). As observed in our growth analyses above, the CFU/ml/ABS_600_ values for the four anaerobic strains did not vary significantly among the cultures (data not shown), demonstrating again that turbidity measurements were an accurate reflection of viable cell counts. Taken together, our results suggest that *hfq∆*/empty vector cells have an intrinsic defect in use of chromium as a terminal electron acceptor during anaerobic respiration.

### The *hfq∆ * mutant is highly sensitive to oxidative stress

**Figure 4:**
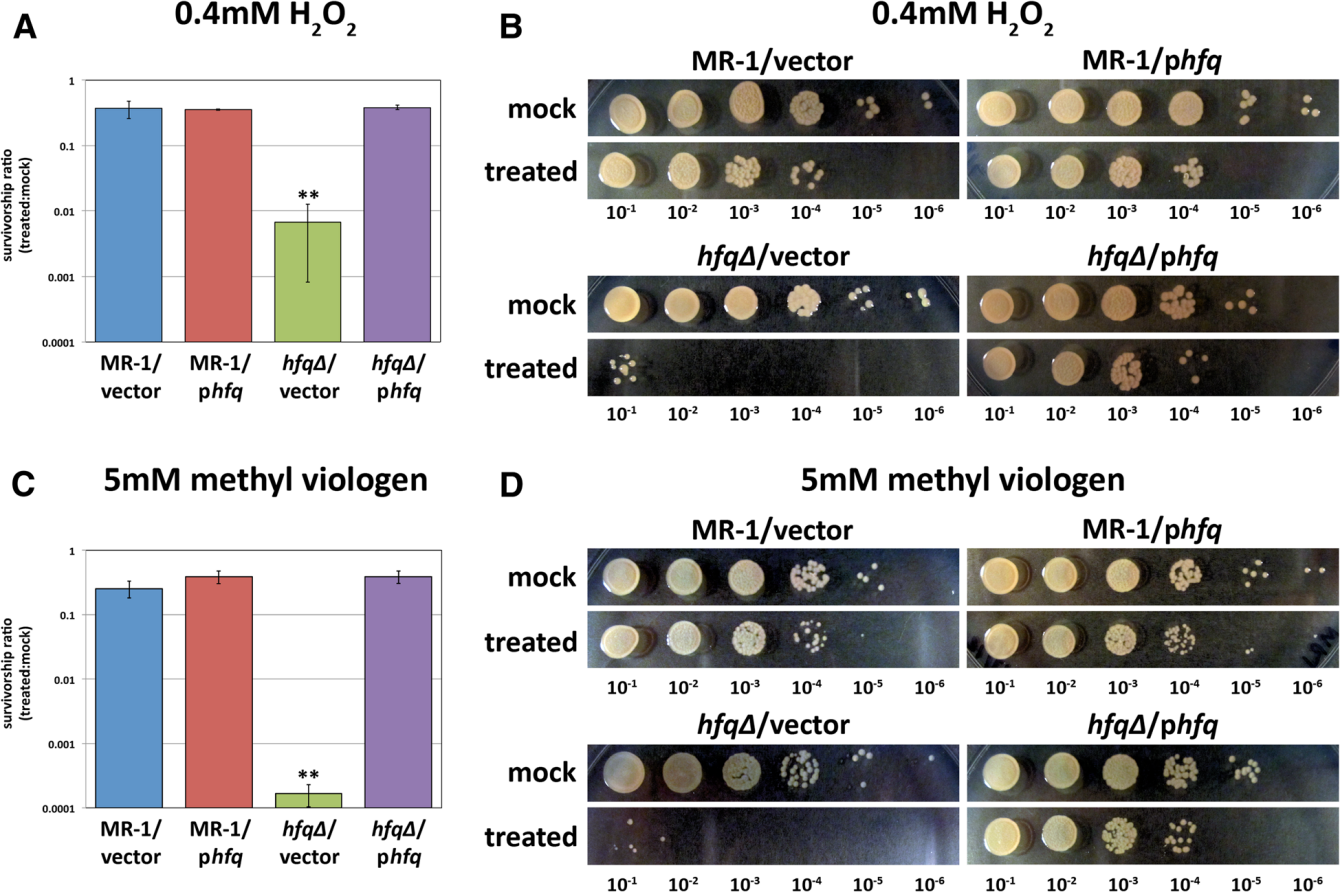
**The *hfq∆ *mutant is highly sensitive to oxidative stress. **Aerobic**,** exponentially growing cultures of MR-1/empty vector, MR-1/p*hfq*, *hfq∆ */empty vector, and *hfq∆ */p*hfq* were treated for 15 minutes with either (**A** and **B**) 0.4 mM H_2_O_2_ or (**C** and **D**) 5mM methyl viologen (paraquat) and then immediately titered. Survivorship ratios were determined by calculating the ratio of the number of viable cells in the treated cultures to the number of viable cells in mock (H_2_O) treated cultures. Values on the graphs are the mean survivorship ratios for three independent experiments. Error bars in (**A**) and (**C**) indicate standard deviations. The *hfq∆ */empty vector survival rate is statistically different from the other three strains in both the H_2_O_2_ and methyl viologen experiments (** indicates that P < 0.005 for comparison of the *hfq∆ */empty vector strain data to each of the other strains in unpaired two-tailed Student’s T-tests). Panels (**B**) and (**D**) demonstrate typical ten-fold dilution series results obtained after treatment of strains MR-1/empty vector, MR-1/p*hfq*, *hfq∆ */empty vector, and *hfq∆ */p*hfq* with (**B**) H_2_O (mock) or H_2_O_2_ or (**D**) H_2_O (mock) or methyl viologen.

Mutations in *hfq* in *E. coli* result in an increased sensitivity to oxidative stress in addition to poor survival in stationary phase [8]. Given the poor survival of the *S. oneidensis hfq∆ * mutant in extended stationary phase, a period typically characterized by increased oxidative stress [22,23], we decided to explore the ability of the *hfq∆ * mutant to cope with oxidative stress. Exponentially growing cultures of MR-1/empty vector, MR-1/p*hfq*, *hfq∆/*empty vector, and *hfq∆*/p*hfq* were treated with either H_2_O_2_ to induce peroxide stress or methyl viologen to induce superoxide stress. Serial dilutions of these cultures were then plated, and the survival rates relative to mock (H_2_O) treated cultures were measured. The survivorship of each strain was determined by calculating the ratio of viable cells in the treated cultures to viable cells in the mock treated cultures. Strains with a wild type copy of *hfq* survived significantly better than the *hfq∆*/empty vector strain when challenged with either H_2_O_2_ (Figure 4A and 4B) or methyl viologen (Figure 4C and 4D). These data suggest that one function of *S. oneidensis* Hfq is to protect cells against oxidative stress. 

## Discussion and conclusions

In this paper, we describe the construction and characterization of a null allele of the *hfq* gene in the bacterium *S. oneidensis*. Loss of the *hfq* gene produces an assortment of phenotypes, each of which is fully complemented by an exogenously supplied copy of the wild type *hfq* gene. To our knowledge, this is the first report of an *hfq* gene knockout in a dissimilatory metal reducing bacterium. Given the varied roles played by Hfq in diverse bacteria, we expect that this mutant will be both a useful tool for analyzing sRNA function in *S. oneidensis* as well as for understanding Hfq function in general.

It is clear from our analyses that *S. oneidensis* Hfq positively regulates exponential phase growth. The exponential phase growth defect of the *hfq* mutant is not growth medium specific, as we observe slow exponential phase growth in both complex and defined media. In addition, we observe this defect when cells are grown under both aerobic and anaerobic conditions. It is not yet clear why the *hfq* mutant grows slowly when nutrients are plentiful. It is possible that the *hfq* mutant growth phenotype is a result of a defect in nutrient acquisition, a possibility suggested by the fact that *hfq* mutants in a variety of bacteria express lower levels of genes involved in nutrient uptake [6,24-26]. It is also possible that the *hfq* mutant has more general set of metabolic defects that underlie its slow growth phenotype, which may explain why the *hfq* mutant is less efficient in Cr(VI) reduction. Alternatively, *hfq* may have a more specific role in utilization of Cr(VI) as a terminal electron acceptor.

A second notable *hfq* mutant growth phenotype is the failure of mutant cultures to achieve a terminal cell density as high as those seen in wild type cultures. Though it is not yet clear what underlies this mutant phenotype, it is possible that the *hfq* mutant is unable to fully utilize the available nutrients in the medium or that it exhausts a nutrient that is rate limiting for growth more rapidly than wild type cells. Alternatively, the *hfq* mutant may produce more of, or be more sensitive to, at least one growth-suppressing product produced during *S. oneidensis* growth.

Strikingly, *S. oneidensis hfq* mutant cultures exhibit a severe loss of colony forming units in stationary phase, with cultures often displaying no detectable CFU. One possibility is that Hfq promotes cell survival in stationary phase, and thus loss of *hfq* results in loss of culture viability. An alternative explanation is that Hfq functions to prevent cells from entering a viable but not culturable (VBNC) state [27], and thus reduced CFU/ml counts in *hfq∆ * mutant cultures are due to *hfq∆ * cells precociously assuming VBNC status. Both of these models are supported by the fact that moderate overexpression of Hfq results in higher CFU/ml counts during stationary phase when compared to cells with wild type Hfq protein levels. Further experimentation will be required to differentiate between these two possible explanations for the greatly reduced CFU/ml counts in *hfq∆ * stationary phase cultures.

Because the *hfq* mutant is highly sensitive to oxidative stress, it is possible that the stationary phase survival defect in *hfq* mutant cells is a consequence of poor resistance to oxidative stress. Multiple Hfq-dependent sRNAs (*arcZ*, *dsrA*, and *rprA*) positively regulate expression of the stationary phase sigma factor RpoS in other systems [28-30]. Thus, it is possible that loss of Hfq in *S. oneidensis* causes low *rpoS* expression, resulting in poor induction of the *rpoS* regulon. Lower *rpoS* regulon induction may increase the oxidative stress sensitivity of the *hfq* mutant and consequently reduce stationary phase survival. Another possibility that remains to be explored is whether the *hfq* mutant’s sensitivity to oxidative stress is due to altered function of superoxide dismutase (*sodB* – So_2881) and/or one or more of the four genes predicted to encode proteins with catalase activity *katB* (So_1070), So_1771.2, *katG2* (So_4405), and *katG1* (So_0725)] [12]. Finally, it will be of interest to determine whether *S. oneidensis* contains an *hfq*-dependent OxyR-OxyS system that is involved in response to oxidative stress as in other systems [20,31].

We are currently investigating the mechanisms by which *S. oneidensis* Hfq promotes growth, terminal culture density, and stationary phase survival. However, given that Hfq has been broadly implicated in the function of many sRNAs in other systems [32], the *S. oneidensis hfq* mutant generated in this study will facilitate analysis of the roles of Hfq and sRNAs in adaptation to a wide range of environmental conditions. This is of particular interest since a previous study demonstrated that *S. oneidensis* sRNAs do not always have completely overlapping functions with their homologs in other systems [33].

## Authors’ contributions

BJP and CMB conceived of and designed all the experiments in the paper, executed experiments, collected and interpreted the data, and drafted the manuscript. Strain construction and verification was performed by BJP, CMB, MLK, TMH, NQM, JMO, KED, MTG, TM, and ZS. BJP and CMB performed stationary phase survival assays and metal reduction assays. BJP, CMB, TMH, MLK, MTG, and NQM designed and performed oxidative stress experiments. All authors read and approved the final manuscript.
